# Physical growth and intelligence development of discordant dizygotic twins from birth to preschool age: a prospective cohort study

**DOI:** 10.1186/s13052-022-01354-y

**Published:** 2022-09-05

**Authors:** Huiqiu Xiang, Xianping Huang, Jing Zhu, Jiajia Chen, Pangpang Zhou, Tong Zhou, Jiale Bao, Zhangye Xu

**Affiliations:** Department of Gynecology and Obstetrics, The Second Affiliated Hospital of Wenzhou Medical University, Wenzhou, 325027 People’s Republic of China

**Keywords:** Discordant growth, Dizygotic twins, Physical development, Intellectual development

## Abstract

**Background:**

The majority of studies are limited to adverse perinatal outcomes and poor cognitive abilities in the short term in discordant monochorionic twins.

**Methods:**

To determine whether small and large discordant dizygotic twins differ in physical growth and intelligence development and weight and height from birth up to 6 years of age were measured in 34 dizygotic twin pairs with ≥ 20% birth weight discordance. Mental developmental index (MDI) and psychomotor developmental index (PDI) were calculated at 1 year, while the Wechsler Intelligence Scale for Children-IV (WISC-IV) full-scale intelligence quotient (IQ) was assessed at the age of 6.

**Results:**

The difference in height and weight in each stage differed significantly from birth to 72-months-old (*P* < 0.05), although there was disappointing catch-up growth in smaller twins. PDI but not MDI at 1 year of age was significantly different between the two groups (*P* < 0.05), and smaller twins experienced higher psychomotor retardation rates (*P* < 0.05). Also, the influence of height and weight on PDI was statistically significant (*P* < 0.05). No significant difference was detected in the WISC-IV full-scale IQ at the age of 6; however, the full-scale IQ may be affected by the history of suffocation and the S/D value (*P* = 0.011, *P* = 0.022).

**Conclusions:**

Intrauterine fetal growth and development lead to birth weight differences in twins and sustain an impact on the children’s physical growth in height and weight from birth to preschool age, causing psychomotor developmental differences at 1 year of age. However, the differences in psychomotor development decrease gradually by the age of 6.

**Supplementary Information:**

The online version contains supplementary material available at 10.1186/s13052-022-01354-y.

## Background

Twin growth discordance is a common finding in multiple gestations. The term describes a significant weight difference between the two fetuses of a twin pregnancy. The definition of significant discordance considered at least a 20% difference in birth weight between the twins [1], although it varies from 15–25% with different cutoffs in clinical practice and research [2, 3]. Twins with ≥ 20% birth weight discordance are associated with adverse pregnancy outcomes, including a high rate of preterm birth, malformation, mortality, neonatal asphyxia, respiratory distress, and admission to the Neonatal Intensive Care Unit (NICU) [4–6]. Nevertheless, data regarding other growth parameters among discordant twins following hospital discharge are lacking. Recent studies have shown that the smaller of the discordant twins after birth attempt to catch up with their siblings in physical growth, thereby significantly reducing the gaps between them during the first year of life [7].

Typically, the prenatal period is the duration of rapid brain development, which includes marked changes in cortical folding [8], myelination [9], and gray-matter distribution [10]. These findings raise the hypothesis that infants who suffer growth restriction during the prenatal period are likely to be deprived of an optimal supply of nutritional substrates and are at risk of impaired neural and cognitive development. Low birth weight is a crucial determinant of psychological development; this the earlier the birth in terms of gestation weeks and the lower the birth weight, the greater the delay in psychological development, especially in the first few years of life [11, 12].

Among healthy twins, growth discordance may be due to the inability of the uterine environment to meet the demands of multiple fetuses, leading to intrauterine growth restriction (IUGR). Twin growth discordance is a complication of both monochorionic and dichorionic twin pregnancies. The etiologies for intrauterine dysplasia in dizygotic twins emerge from maternal and fetal factors, while in monozygotic twins, non-intrauterine nutrition factors, such as twin transfusion syndrome (TTTS) and selective IUGR (sIUGR), underlie poor prognosis. Chorionicity represents a putative confounder in the prevalence of neurologic morbidity among twin survivors. The current studies on postnatal physical and mental development in discordant twins have not distinguished the chorion, which could skew the results. Therefore, the present study discusses the physical growth and intelligence development of discordant dizygotic twins. Can the smaller of the discordant twins catch up with their sibling in physical and mental development after birth?

## Methods

### Subjects

The study included all neonatal discordance dizygotic twins born at The Second Affiliated Hospital of Wenzhou Medical University (WMU) from June 2012 to June 2013 and survived to discharge. Dizygotic twins whose birth weight differed by ≥ 20% were classified as discordant twins. Informed consent was obtained from the parents before enrollment in the study. The Second Affiliated Hospital of WMU Ethics Committee approved the study. The exclusion criteria were infants with major congenital malformations, such as major cardiac malformations, chromosomal anomalies, and skeletal, renal, brain, and lung malformations. According to the birth weight in each twin pair, the smaller one was categorized into Group Twin-Low birthweight (Group Twin-L), and the larger one was assigned to Group Twin-High birthweight (Group Twin-H).

### Procedure

Information related to maternal (maternal ethnicity, weight, height, age, marital status, education, family income, health insurance status, social status, reproductive history, and medical diseases before pregnancy) and neonatal (including pregnancy systolic/diastolic peak (S/D) value, birth weight, length, head circumference, gestational age, gender, malformation, Apgar score, respiratory distress, NICU entry, and feeding option) characteristics were collected from the medical files of the mothers and infants or an in‐person interview.

The height and weight parameters of each child were measured by physical examinations or collected from the Wenzhou Maternal and Child Healthcare system at birth and 1-, 3-, 6-, 8-, 12-, 18-, 30-, 36-, 48-, 60-, and 72-months-old. The electronic baby scale and clinostatic body length standard bed were designed to help monitor the weight and body length (measured lying flat with a single suit) of children < 36-months-old. A uniform height and weight scale were designed to help monitor the height and weight gain (measured standing without shoes) for children aged ≥ 36 months. All measurements were performed by the same experienced physician.

Bayley Scales of Infant Development (BSID), including intelligence and exercise scales, was used to test the psychomotor and mental development of 1-year-old infants. The intelligence and exercise scales were expressed by mental development index (MDI) and psychomotor development index (PDI), respectively. After the infants gained familiarity and were cooperative in a quiet environment, the evaluation process was carried out item by item to estimate the MDI and PDI. The MDI and PDI scores were defined as follows: low (< 70), critical (70–89), moderate (90–109), and high (≥ 110).

Wechsler Intelligence Scale for Children-IV (WISC-IV), including verbal test and operational test, was used to assess the intelligence development of 6-year-old children. After obtaining full familiarity and cooperation from the children in a quiet environment, the evaluation process was carried out item by item to deduce the full-scale IQ, including verbal comprehension index (VCI), perceptual reasoning index (PRI), working memory index (WMI), and processing speed index (PSI). According to the scores of the full-scale IQ, children were distributed into two groups: Group IQ-Normal (IQ-N) with scores ≥ 90 and Group IQ-Low (IQ-L) with scores < 90.

Cognitive performance tests were carried out by expert psychologists (who had a degree of Master of Arts in Psychology) at the Institute of Cognitive Sciences of the Second Affiliated Hospital of WMU. The examiners were fully unaware of the status of the twins. The evaluation was repeated after 15 days if the children did not cooperate satisfactorily.

### Statistical analysis

The smooth spline function of R 3.5.3 was used to fit the sample data with three smoothing splines. Firstly, the parameter “CV (cross-validation) = true” was set to select the appropriate degree of freedom for the group with a small sample size using the leave-one-out cross-validation. Then, the fitting curves of the physical indicators of each group were drawn based on this degree of freedom. The data were analyzed using SPSS 19.0 software. Paired t-test was used to compare the measured data between normal distribution datasets, expressed as mean ± standard deviation, while paired Wilcoxon rank-sum test was used to compare the non-normal data sets, expressed as (minimum, maximum). The chi-square test was used for enumeration data, expressed as a ratio or constituent ratio. In univariate analysis, the t-test was used to compare the normal distribution variables, the Mann–Whitney U test for skewed distribution variables, and Fisher’s exact test was utilized for classified variables. In multivariate analysis, the linear regression method was applied. *P* < 0.05 indicated a statistically significant difference. The predictive performance of dependent variables to predict the degree of IQ scores was quantified using the area under the curve (AUC) of the receiver operating characteristic (ROC) analysis. An AUC of 0.5 represents a variable with no predictive ability, and the closer the AUC to 1.0, the higher the authenticity of the variable.

## Results

### Basic information

During the study period, 58 sets of discordance dizygotic twins were born at the Second Affiliated Hospital of WMU and survived to discharge. Of these, 6 pairs were unavailable to contact, 16 did not consent, 1 pair in whom the smaller twin was confirmed to have cerebral palsy, and 1 pair in whom the smaller twin suffered congenital heart disease were excluded. Finally, we obtained a total of 34 pairs with a 58.6% inclusion rate. All children accepted chromosomal analysis and medical tests. No chromosomal abnormalities, skeletal, renal, brain, and lung malformations was found. The mean gestational age at birth was 35 ± 1.86 (30–38) weeks. The mean birth weight difference was 738 ± 285.89 g (300–1380 g), and the mean percentage difference in birth weight was 28% (20–50%).

### Physical development from birth to 6-years-old

**Fig. 1:**
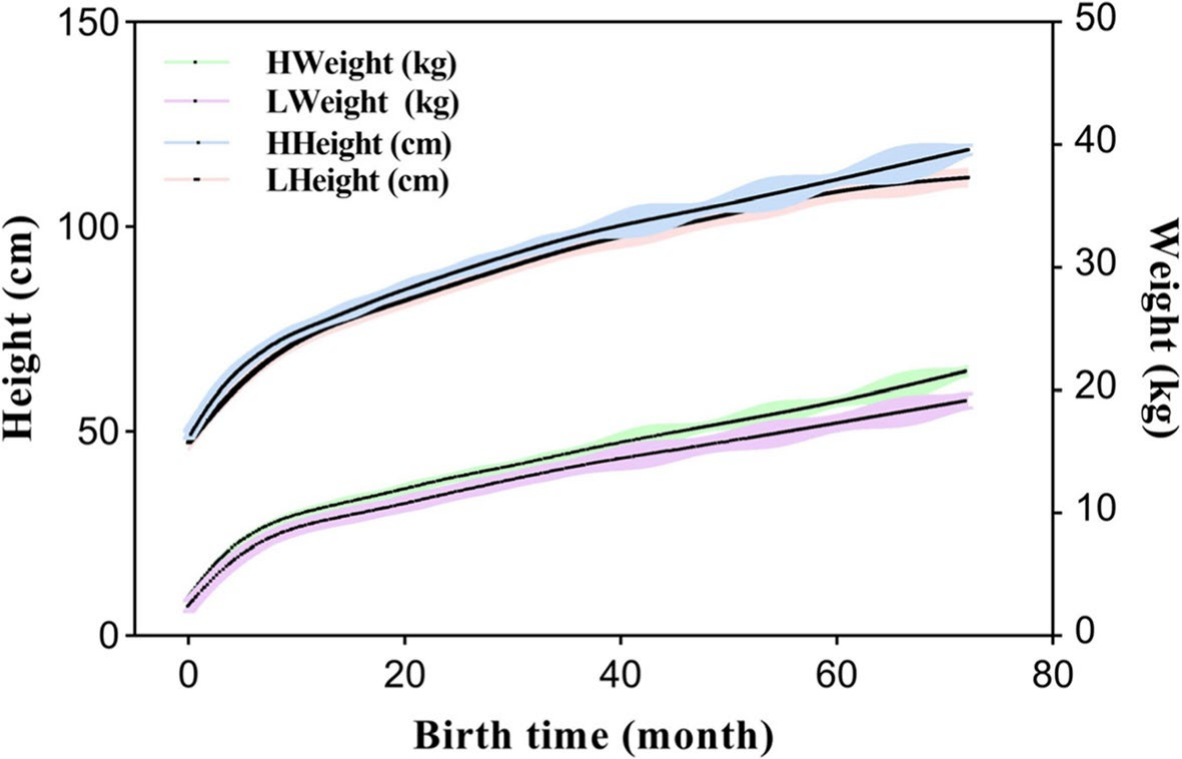
The fitting curves of the physical indicators in Twin-H and Twin-L. HWeight: weights in group of Twin-High birthweight; LWeight: weights in the group of Twin-Low birthweight; HHeight: heights in group of Twin-High birthweight; LHeight: heights in the group of Twin-Low birthweight

The physical development data of 34 discordant dizygotic twin pairs at birth and 1, 3-, 6-, 8-, 12-, 18-, 30-, 36-, 48-, 60-, and 72-months-old were reviewed, and the fitting curves of body weight and height were drawn, respectively (Fig. 1). The height and weight of Group Twin-L children did not catch up with Group Twin-H at each stage. In addition, the difference in body weight and height between Twin-L and Twin-H showed a stable trend in the first year of life without significant narrowing. The physical development data are described and compared in Table 1. The height and weight of Group Twin-L children were significantly lower than those of Group Twin-H children at all time points within 72 months (*P* < 0.05). The stratified analysis further demonstrated that the weight growth rate of Group Twin-L in 0–1, 3–6 months, and 24–30 months, and height growth rate in 1–3 and 8–12 months were significantly higher than those of Group Twin-H (*P* < 0.05).

**Table 1:** Comparison between Twin-H and Twin-L in weight (kg) and height (cm) and growth rates (%)

Time	H-Weight	L-Weight	*P*	H-Height	L-Height	*P*	H-Weight growth rate	L-Weight growth rate	*P*	H-Height growth rate	L-Height growth rate	*P*
Birth	2.8 ± 0.5	2.0 ± 0.4	< 0.001	50.0 (48.0,50.0)	47.0 (42.0,48.0)	< 0.001						
1 month	4.4 ± 0.7	3.6 ± 0.6	< 0.001	54.3 ± 2.3	51.0 ± 3.0	< 0.001	54.8 (37.6,72.4)	74.2 (57.9,105.9)	< 0.001	12.4 ± 5.9	13.1 ± 5.4	0.482
3 months	6.5 ± 0.8	5.5 ± 0.8	< 0.001	61.3 ± 2.4	58.7 ± 3.3	< 0.001	51.5 ± 21.8	57.8 ± 24.7	0.086	13.0 ± 4.2	15.2 ± 4.5	0.006
6 months	8.3 ± 1.0	7.2 ± 1.0	< 0.001	68.2 (67.0,69.0)	65.4 (63.5,67.0)	< 0.001	25.3 (18.2,32.3)	28.6 (20.5,40.5)	0.013	10.5 (7.2,13.6)	10.0 (8.2,13.3)	0.596
8 months	9.3 ± 0.8	8.2 ± 1.0	< 0.001	71.9 ± 3.0	69.2 ± 3.4	< 0.001	12.6 ± 7.6	14.6 ± 9.4	0.072	5.8 (4.0,7.0)	5.9 (4.6,7.5)	0.456
12 months	10.3 ± 1.0	9.2 ± 1.0	< 0.001	76.4 ± 2.6	74.2 ± 3.0	< 0.001	11.2 ± 7.4	12.3 ± 6.0	0.381	6.2 ± 2.7	7.3 ± 2.6	0.012
18 months	11.5 ± 1.1	10.3 ± 1.1	< 0.001	82.8 ± 2.8	79.7 ± 3.5	< 0.001	12.3 ± 7.4	12.8 ± 7.5	0.635	8.5 ± 3.0	7.4 ± 3.2	0.115
24 months	12.8 ± 1.2	11.6 ± 1.3	< 0.001	89.0 (87.0,90.0)	86.0 (85.0,88.0)	< 0.001	11.5 ± 5.0	12.4 ± 5.9	0.351	6.7 ± 2.1	7.3 ± 2.4	0.309
30 months	13.9 ± 1.3	12.7 ± 1.4	< 0.001	93.5 ± 3.2	90.3 ± 3.9	< 0.001	8.0 ± 4.7	9.8 ± 5.3	0.035	5.7 ± 2.4	5.6 ± 2.2	0.838
36 months	15.1 ± 1.4	13.8 ± 1.5	< 0.001	98.0 (96.0,100.0)	96.0 (93.5,98.0)	< 0.001	8.8 ± 4.9	8.9 ± 5.3	0.911	5.2 (3.2,6.5)	5.3 (4.3,6.6)	0.092
48 months	17.1 ± 1.6	15.6 ± 1.8	< 0.001	104.0 (102.0,107.0)	102.0 (99.0,104.0)	< 0.001	13.6 ± 4.8	12.6 ± 6.9	0.432	6.8 (5.2,8.0)	6.1 (5.1,8.5)	0.879
60 months	19.1 ± 2.1	17.4 ± 2.2	< 0.001	111.6 ± 4.6	108.7 ± 5.0	< 0.001	11.7 ± 4.9	11.3 ± 5.4	0.735	6.5 (4.9,7.8)	6.5 (4.9,7.8)	0.798
72 months	21.5 ± 2.5	19.7 ± 2.3	< 0.001	118.8 ± 5.0	115.2 ± 5.6	< 0.001	11.6 (8.0,15.8)	12.6 (8.7,16.7)	0.848	6.5 ± 1.9	6.0 ± 1.9	0.009

*H-Weight* Weights in group of Twin-High birthweight, *L-Weight* Weights in the group of Twin-Low birthweight, *H-Height* Heights in group of Twin-High birthweight, *L-Height* Heights in the group of Twin- Low birthweight, growth rate = (indicators in this month-indicators in last month)/indicators in this month

### Intelligence development at 1- and 6-years-old

The smaller twin presented low PDI scores than the larger at the age of 1 (*P* < 0.05); however, no significant difference was observed in MDI at the age of 1, and all the four index scores comprised the full-scale IQ at 6 years of age between the two groups (*P* > 0.05) (Table 2). The psychomotor retardation rates (PDI < 70) were 17.6% in Group Twin-L and 2.9 in Group Twin-H, indicating a significant difference between the two groups (*P* < 0.05) (Table 3). However, no significant difference was noted in the mental retardation rates (MDI < 70) between the two groups (*P* > 0.05, 17.6% in Group Twin-L and 11.8% in Group Twin-H).

**Table 2:** BSID results and WISC-IV results for Group Twin-L *vs*. Group Twin-H

Group	Twin-H	Twin-L	Paired-T	*P*
PDI	104.76 ± 15.81	96.09 ± 19.09	4.239	< 0.001
MDI	94.29 ± 19.84	90.26 ± 19.23	1.66	0.106
VCI	95.60 ± 9.41	94.94 ± 7.96	0.344	0.733
PRI	103.58 ± 11.87	99.61 ± 15.25	1.701	0.099
WMI	95.27 ± 10.97	94.73 ± 10.63	0.269	0.789
PSI	99.48 ± 10.87	97.55 ± 10.91	0.959	0.345
Full-scale IQ	97.94 ± 7.72	95.85 ± 9.29	1.383	0.176

*Twin-H* The group of Twin-High birthweight, *Twin-L* The group of Twin- Low birth weight

*PDI* Psychomotor development index, *MDI* Mental development index, *VCI* Verbal comprehension index, *PRI* Perceptual reasoning index, *WMI* Working memory index, *PSI* Processing speed index, *IQ* Intelligence quotient

**Table 3:** Psychomotor and mental retardation rates for Group Twin-L *vs*. Group Twin-H

Scores	Level	PDI		MDI	
		Twin-H	Twin-L	Twin-H	Twin-L
< 70	Lower	1 (2.9) ^a^	6 (17.6)	4 (11.8)	6 (17.6)
70–89	Intermediate	6 (17.5)	6 (17.5)	8 (23.5)	8 (23.5)
90–109	Medium	14 (41.2)	12 (35.3)	14 (41.2)	17 (50.0)
≥ 110	Excellent	13 (38.2)	10 (29.4)	8 (23.5)	3 (8.8)

*Twin-H* The group of Twin-High birthweight, *Twin-L* The group of Twin- Low birth weight

*PDI* Psychomotor development index, *MDI* Mental development index

^a^Psychomotor retardation rates (PDI < 70) in Group Twin-L were significantly higher than in Group Twin-H, *P* < 0.05

### Multiple linear regression analysis in 1-year-olds

A total of 68 cases of MDI and PDI were analyzed by multiple linear regression severally. PDI and MDI were taken as dependent variables, and the main feeder’s education degree, age, feeding option, weight, height, gender, gestational age, birth weight, S/D value, asphyxia (Apgar score ≤ 7), and infection were considered independent variables. The effects of body height and weight on were statistically significant on PDI (*F* = 11.40, *P* = 0.0012; *F* = 6.21, *P* = 0.0153), but not on MDI (*F* = 0.95, *P* = 0.3350; *F* = 1.04, *P* = 0.3137) in supplementary table 2. The stepwise regression analysis showed that the effects of weight on PDI were statistically significant (*P* < 0.05), but the representativeness of individual indicators was insufficient (*R*^*2*^ = 0.16), as supplementary table 3 shown. Moreover, the differences in the effects of other factors, such as asphyxia and S/D value, PDI, and MDI, were not statistically significant (*P* > 0.05).

### IQ analysis in 6-year-olds

**Fig. 2:**
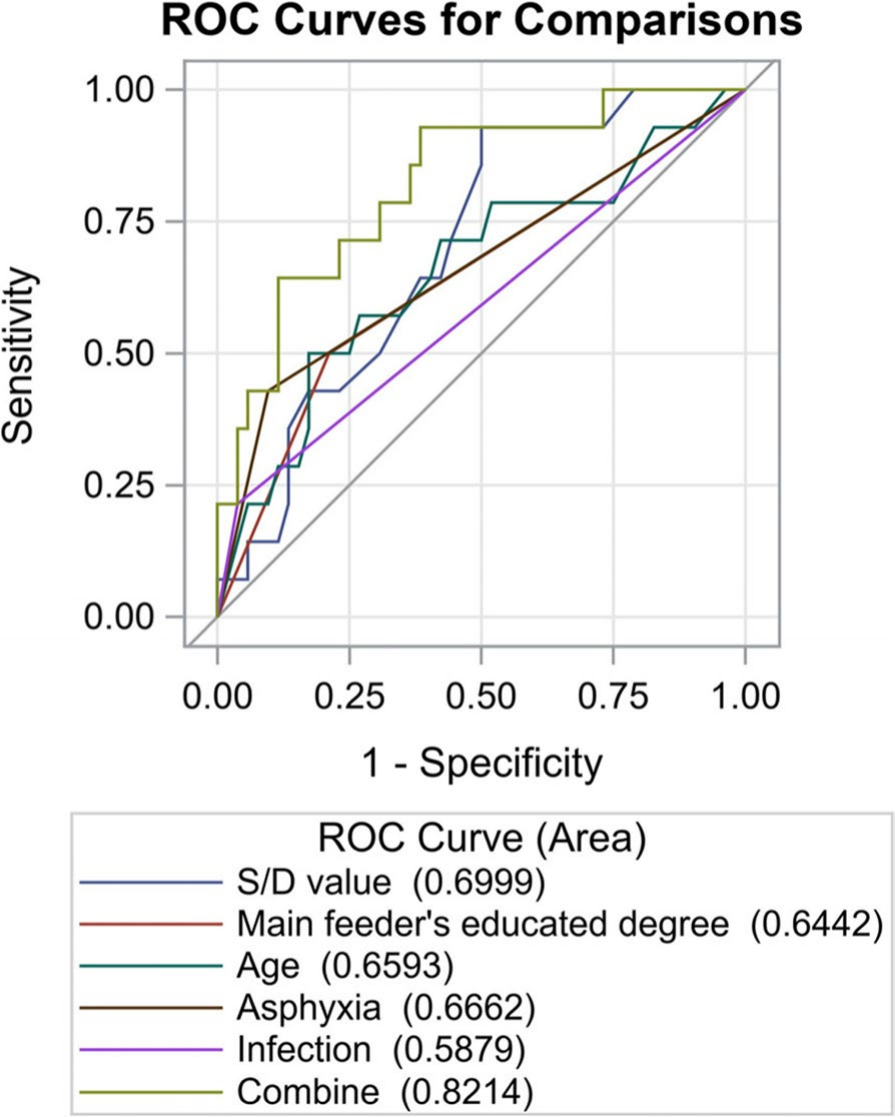
The AUC of S/D value, main feeder’s education degree, age, asphyxia, infection, and combination

No statistically significant factors contributing to low IQ scores were identified by multiple linear regression analysis. Thus, the independent variables of 68 children in the IQ-N group (full-scale IQ scores ≥ 90) and IQ-L group (full-scale IQ scores < 90) are described (Table 4). We found that the asphyxia rate and S/D value of the IQ-N group (9.4%, 2.3 (2.0, 2.8)) were lower than that of the IQ-L group (40.0%, 2.8 (2.4, 3.1)) (*P* < 0.05). Univariate analysis (supplementary table 1) identified the independent variables with *P* < 0.1, including S/D value (*P* = 0.081), age (*P* = 0.071), education degree of the main feeder (*P* = 0.038), asphyxia (*P* = 0.006), and infection (*P* = 0.048), and a ROC curve was drawn (Fig. 2). The maximum AUC of the S/D value was 0.6999, indicating that the IQ was largely related to it.

**Table 4:** Several factors distributed in IQ-L *vs*. IQ-N

Factor	IQ-L	IQ-N	*P*
Gestational age (weeks)	35.9 (34.0,36.8)	36.2 (34.6,37.0)	0.604
S/D value	2.8 (2.4,3.1)	2.3 (2.0,2.8)	0.022
Birth weight (g)	2116.4 ± 573.7	2302.3 ± 539.8	0.263
Age	6.7 ± 0.7	6.4 ± 0.7	0.065
Body weight (kg)	22.6 ± 5.9	23.6 ± 4.4	0.500
Body height (cm)	123.3 ± 8.1	124.4 ± 7.0	0.618
Educated degree			0.07
≤ junior high school	8 (50.0)	11 (21.2)	
≥ senior high school	8 (50.0)	41 (78.8)	
	16	52	
Gender			0.635
girl	6 (42.9)	27 (50.0)	
boy	8 (57.1)	27 (50.0)	
	14	54	
Feeding option			0.054
artificial feeding	14 (100.0)	38 (70.3)	
breast feeding	0 (0.0)	16 (29.7)	
	14	54	
Asphyxia			0.011
no	9 (60.0)	48 (90.6)	
yes	6 (40.0)	5 (9.4)	
	15	53	
Infection			0.101
no	12 (80.0)	51 (96.2)	
yes	3 (20.0)	2 (3.8)	
	15	53	

*IQ-L* The group with the IQ scores < 90, *IQ-N* The group with the IQ scores ≥ 90

## Discussion

Several studies have shown a strong association of low birth weight to adult growth and health [11, 12]. Birth weight can be seen as an index of fetal nutrition, influenced by maternal factors and fetal genetics [13, 14], the latter acting through the intrauterine environment. A twin study model is an insightful means to control maternal factors; however, the data describing the growth patterns among discordant twins are limited and whether the smaller twins present a worse outcome than their larger counterparts in adult life is debatable. Mazkereth R et al. [15] showed that the gaps in growth parameters between the smaller the discordant twins and their larger siblings are significantly decreased during the first year of life. Ross GS et al. [16] suggested that the smaller the discordant twins exhibited an appropriate catch-up, resulting in no differences in any growth parameters at the age of 3; nonetheless, only 16 pairs of discordant twins were assessed, and the mean gestational age was 31 ± 2.4 weeks. The current study indicated that the difference in birth weight of dizygotic twins had a lasting effect on the physical growth in infancy, and the smaller had no obvious catch-up growth from birth to 6 years. Next, we measured the growth rates of discordant dizygotic twins at different time points and found that the smaller of the discordant twins, except for the one that gained weight quickly in the first 6 months after birth, did not catch up with the larger twins. Regarding height, there were no rules in the growth rate pattern. We suggested the follow-up physical examination should be required for discordant twins in the future, especially for the smaller one, to guard against short statures, malnutrition and growth retardation in adult. Some studies demonstrated that the twins attained normal final physical development compared to children from the general population until late adolescence [17, 18]. Thus, monitoring these discordant dizygotic twins is recommended to observe whether they have narrowed the gap in physical parameters during adolescence.

Low birth weight and prenatal malnutrition are associated with neurodevelopmental disorders [19, 20]; however, the exact mechanism is yet to be clarified. A previous study speculated that the adverse intrauterine environment leads to the hypothalamus-pituitary-adrenal axis resetting, and long-term stimulation of glucocorticoids damages the microvascular endothelial cells, resulting in nerve injury in infants with IUGR [21]. The scarcity of uterine resources restricts intrauterine growth, which has a detrimental effect on cognitive development in childhood. The intelligence development level of the same twin pairs was measured at ages 1 and 6. The smaller twins performed significantly worse in psychomotor skills but not cognition at the age of 1, and their psychomotor retardation rates were 17.6% compared to the larger twins (2.9%). Birth weight growth discordance of ≥20% confers an independent adverse effect on the long-term neurodevelopment of the smaller twin. According to the results of multiple linear regression analysis on psychomotor development, we proposed that delayed psychomotor development was associated with small body size during infancy, as described previously by Silventoinen K et al. [22] In addition, Halling C et al. [23] considered birth weight growth as an independent adverse effect on the long-term neurodevelopment and demonstrated a negative impact of growth discordance on the smaller twin at the age of 2.5 years across all three domains of development: motor, language, and cognition. We suggested discordant twins should be tested with BSID routinely at one year old, so as to give access to early clinical intervention for the smaller infants with psychomotor retardation.

The gap in intelligence development in discordant dizygotic twins at the age of 6 appears to be bridged because no significant difference was detected in VCI, PRI, WMI, PSI, and the full-scale IQ in our study. However, smaller twins had a PRI score 4 points lower than the larger twin (Twin-L 99.61 ± 15.25 *vs*. Twin-H 103.58 ± 11.87). Thus, we suspected that the wide PRI score difference could be attributed to the gap in PDI at the age of 1. The smaller twins accepted preschool education inevitably under pressure from parents, who wanted to narrow the gap after the BSID assessment. Some studies on the monochorionic twin cohort [24, 25] reported fetal growth restriction results in lower neurocognitive scores in early childhood and significant differences in size.

Typically, the general indicators can affect IQ scores, among which the prenatal environment is discussed previously [26]. The high pregnancy S/D value can predict fetal intrauterine distress and poor perinatal outcomes [27]; however, whether subtle variations in the routine ultrasound detection are associated with long-term cognitive and motor abilities is yet to be elucidated. The occurrence of fetal distress might lead to neonatal asphyxia. Chronic asphyxia can cause cerebral and hypoxia, affecting the children’s quality of later life. We found that the IQ-L group had a higher pregnancy S/D value and perinatal asphyxia rate than the IQ-H group. The current study carried out univariate analysis and multivariate linear regression analysis to control the confounding factors, and the ROC curve was drawn for an intuitive picture of the possible factors affecting the IQ. The pregnancy S/D value comprised the maximum AUC, followed by asphyxia, pointing to a link with IQ. Therefore, additional clinical cases will be collected in future studies to verify the influence of pregnancy S/D value and neonatal asphyxia on children’s IQ scores. As the smaller twins’ body size grew with age, the basic and complex skills improved, especially fine motion abilities. This could explain the association of body weight with intelligence level at the age of 1, although the effect diminished at the age of 6.

Nevertheless, we did not analyze other physical indicators, such as head, chest, and abdomen circumferences. The assessments of intelligence development were only performed on 1- and 7-year-olds due to the lack of cooperation from children. Furthermore, we could not keep track of all the twins to assess whether they have narrowed the gap in physical parameters during adolescence.

## Conclusions

In summary, birth weight differences in twins sustain an impact on the children’s physical growth in height and weight from birth to preschool age, causing psychomotor developmental differences at the age of 1. However, the differences in psychomotor development narrowed gradually at the age of 6.

## Supplementary Information

**Additional file 1: Supplementary Table 1.** Univariate analysis in IQ-L *vs*. IQ-N. We used univariate analysis to identify the independent variables with *P*<0.1 in the IQ-N group (full-scale IQ scores ≥90) and IQ-L group (full-scale IQ scores <90). **Supplementary Table 2.** Multiple linear regression analysis at 1-year-old. The MDI and PDI were analyzed by multiple linear regression. PDI and MDI were taken as dependent variables, and the main feeder’s education degree, age, feeding option, weight, height, gender, gestational age, birth weight, S/D value, asphyxia (Apgar score ≤7), and infection were considered independent variables. **Supplementary Table 3.** Effect of weight on PDI by stepwise regression analysis. The stepwise regression analysis showed that the effects of weight on PDI were statistically significant (*P*<0.05), but the representativeness of individual indicators was insufficient (*R*^*2*^=0.16).

## Data Availability

All data generated or analyzed during this study are included in this published article.

## Abbreviations

MDI: Mental Developmental Index
PDI: Psychomotor Developmental Index
WISC-IV: Wechsler Intelligence Scale for Children-IV
IQ: Intelligence Quotient
NICU: Neonatal Intensive Care Unit
MC: Monochorionic
DC: Dichorionic
TTTS: Twin Transfusion Syndrome
sIUGR: Selective Intrauterine Growth Restriction
WMU: Wenzhou Medical University
S/D: Systolic/Diastolic peak
BSID: Bayley Scales of Infant Development
ROC: Receiver Operating Characteristic
AUC: Area Under the Curve
VCI: Verbal Comprehension Index
PRI: Perceptual Reasoning Index
WMI: Working Memory Index
PSI: Processing Speed Index
