# Supplementary material for: Physical growth and intelligence development of discordant dizygotic twins from birth to preschool age: a prospective cohort study
Source: Ital J Pediatr. 2022 Sep 5;48:162. doi: 10.1186/s13052-022-01354-y (PMC9446820; doi:10.1186/s13052-022-01354-y)
Supplement: Supplementary file 1 — Additional file 1: Supplementary Table 1. Univariate analysis in IQ-L vs. IQ-N. We used univariate analysis to identify the independent variables with P<0.1 in the IQ-N group (full-scale IQ scores ≥90) and IQ-L group (full-scale IQ scores <90). Supplementary Table 2. Multiple linear regression analysis at 1-year-old. The MDI and PDI were analyzed by multiple linear regression. PDI and MDI were taken as dependent variables, and the main feeder’s education degree, age, feeding option, weight, height, gender, gestational age, birth weight, S/D value, asphyxia (Apgar score ≤7), and infection were considered independent variables. Supplementary Table 3. Effect of weight on PDI by stepwise regression analysis. The stepwise regression analysis showed that the effects of weight on PDI were statistically significant (P<0.05), but the representativeness of individual indicators was insufficient (R2=0.16). [file 13052_2022_1354_MOESM1_ESM.docx]

**Supplementary Table 1** Univariate analysis in IQ-L *vs*. IQ-N

| **Variable** | **OR(95% CI)** | ***P*-value** | **AOR (95% CI)** | ***P*-value** |
| --- | --- | --- | --- | --- |
| Gestational age (week) | 0.992(0.721–1.363) | 0.958 |  |  |
| S/D value | 0.465(0.197–1.099) | 0.081 | 1.898(0.585–6.162) | 0.286 |
| Birth weight (g) | 1.001(1.000–1.002) | 0.261 |  |  |
| Age(month) | 0.409(0.155–1.080) | 0.071 | 2.921(0.925–9.224) | 0.068 |
| Body weight (kg) | 1.048(0.916–1.199) | 0.494 |  |  |
| Body height (cm) | 1.021(0.942–1.108) | 0.613 |  |  |
| Educated degree |  |  |  |  |
| ≤junior high school | 1.000(1.000–1.000) | Ref. | 1.000(1.000–1.000) | Ref. |
| ≥senior high school | 3.727(1.077–12.895) | 0.038 | 0.357(0.08–1.59) | 0.177 |
| Gender |  |  |  |  |
| girl | 1.000(1.000–1.000) | Ref. |  |  |
| boy | 0.750(0.228–2.465) | 0.636 |  |  |
| Feeding option |  |  |  |  |
| Artificial feeding | 1.000(1.000–1.000) | Ref. |  |  |
| breast feeding | - | - |  |  |
| Asphyxia |  |  |  |  |
| no | 1.000(1.000–1.000) | Ref. | 1.000(1.000–1.000) | Ref. |
| yes | 0.142(0.035–0.577) | 0.006 | 4.787(0.896–25.587) | 0.067 |
| Infection |  |  |  |  |
| no | 1.000(1.000–1.000) | Ref. | 1.000(1.000–1.000) | Ref. |
| yes | 0.147(0.022–0.985) | 0.048 | 3.745(0.37–37.906) | 0.264 |

IQ-L: the group with the IQ scores <90; IQ-N: the group with the IQ scores ≥90; OR: odds ratio; AOR: adjusted odds ratio.

**Supplementary Table 2** Multiple linear regression analysis at 1-year-old

| **Variable** | PDI | | | MDI | | |
| --- | --- | --- | --- | --- | --- | --- |
|  | Mean Square | F Value | Pr > F | Mean Square | F value | Pr > F |
| Gestational Age (weeks) | 36.688 | 0.14 | 0.7089 | 789.901 | 2.24 | 0.1422 |
| S/D value | 136.396 | 0.53 | 0.4726 | 0.039052 | 0.00 | 0.9917 |
| Birth weight(g) | 36.884 | 0.14 | 0.7081 | 55.122 | 0.16 | 0.6947 |
| Age | 759.619 | 2.93 | 0.0947 | 848.587 | 2.41 | 0.1286 |
| Body weight (kg) | 1946.8586 | 6.21 | 0.0153 | 269.957 | 1.04 | 0.3137 |
| Body height (cm) | 3571.652 | 11.4 | 0.0012 | 246.994 | 0.95 | 0.335 |
| Education degree | 137.822 | 0.53 | 0.4703 | 139.112 | 0.39 | 0.5335 |
| Gender | 888.918 | 2.861 | 0.095 | 383.446 | 1.06 | 0.307 |
| Feeding option | 0.567 | 0.02 | 0.967 | 1.037 | 0.00 | 0.958 |
| Asphyxia | 375.738 | 1.45 | 0.2358 | 1040.395 | 2.95 | 0.0935 |
| Infection | 118.863 | 0.369 | 0.546 | 5.471 | 0.37 | 0.546 |

**Supplementary Table 3** Effect of weight on PDI by stepwise regression analysis

| **Variable** | DF | Mean square | F value | R^2^ | Pr > F |
| --- | --- | --- | --- | --- | --- |
| Body weight (kg) | 1 | 5008.380 | 20.17 | 0.16 | 0.0001 |
